# Estimating mandibular growth stage based on cervical vertebral maturation in lateral cephalometric radiographs using artificial intelligence

**DOI:** 10.1186/s40510-024-00527-1

**Published:** 2024-06-24

**Authors:** Sajjad Alipour Shoari, Seyed Vahid Sadrolashrafi, Aydin Sohrabi, Reza Afrouzian, Pooya Ebrahimi, Maryam Kouhsoltani, Minou Kouh Soltani

**Affiliations:** 1 Department of Orthodontics, Faculty of Dentistry, Tabriz University of Medical Sciences, Tabriz, Iran; 2 Mianeh Technical and Engineering Faculty, University of Tabriz, Tabriz, 51666-14766 Iran; 3 Department of Orthodontics, Tabriz University of Medical Sciences, Tabriz, Iran; 4 Department of Oral and Maxillofacial Pathology, Faculty of Dentistry, Tabriz University of Medical Sciences, Tabriz, Iran

**Keywords:** Cervical vertebrae maturation, Mandible growth spurt, Artificial intelligence, Transfer learning, Orthodontics

## Abstract

**Introduction:**

Determining the right time for orthodontic treatment is one of the most important factors affecting the treatment plan and its outcome. The aim of this study is to estimate the mandibular growth stage based on cervical vertebral maturation (CVM) in lateral cephalometric radiographs using artificial intelligence. Unlike previous studies, which use conventional CVM stage naming, our proposed method directly correlates cervical vertebrae with mandibular growth slope.

**Methods and materials:**

To conduct this study, first, information of people achieved in American Association of Orthodontics Foundation (AAOF) growth centers was assessed and after considering the entry and exit criteria, a total of 200 people, 108 women and 92 men, were included in the study. Then, the length of the mandible in the lateral cephalometric radiographs that were taken serially from the patients was calculated. The corresponding graphs were labeled based on the growth rate of the mandible in 3 stages; before the growth peak of puberty (pre-pubertal), during the growth peak of puberty (pubertal) and after the growth peak of puberty (post-pubertal). A total of 663 images were selected for evaluation using artificial intelligence. These images were evaluated with different deep learning-based artificial intelligence models considering the diagnostic measures of sensitivity, specificity, accuracy, positive predictive value (PPV), and negative predictive value (NPV). We also employed weighted kappa statistics.

**Results:**

In the diagnosis of pre-pubertal stage, the convolutional neural network (CNN) designed for this study has the higher sensitivity and NPV (0.84, 0.91 respectively) compared to ResNet-18 model. The ResNet-18 model had better performance in other diagnostic measures of the pre-pubertal stage and all measures in the pubertal and post-pubertal stages. The highest overall diagnostic accuracy was also obtained using ResNet-18 model with the amount of 87.5% compared to 81% in designed CNN.

**Conclusion:**

The artificial intelligence model trained in this study can receive images of cervical vertebrae and predict mandibular growth status by classifying it into one of three groups; before the growth spurt (pre-pubertal), during the growth spurt (pubertal), and after the growth spurt (post-pubertal). The highest accuracy is in post-pubertal stage with the designed networks.

## Background

Dentofacial deformities are one of the problems related to the jaw that can affect different aspects of a person’s life. The task of treating dentofacial deformities is the responsibility of orthodontics. Many treatment methods will yield in better results in shorter time if they are properly correlated with the growth patterns of a patient [1]. Growth-related devices such as functional appliances, and extra-oral elastics (facemask, headgear) should be used during the growth spurt period. Also, jaw surgery can only be performed after the end of puberty, because significant growth after that may cause recurrence [2]. As a result, determining the amount of residual growth of the mandible has an effect on diagnosis, prognosis, and treatment.

Growth spurt time can vary from 2 to 3 years on each side according to the average chronological age. Apparently, the chronological age is not suitable for assessing the developmental stages [2]. One of the approaches to estimating growth spurt is the use of cervical vertebral maturation (CVM) stages using lateral cephalometric images. This method is based on vertebras morphology. Researchers have classified different stages of CVM and related them to the growth slope of the mandible [3]. Previous studies on the relationship between the stages of CVM and the growth spurt of the mandible have expressed contradictory results. Some of them show poor [4–6] and some others display intermediate [7, 8] relation. Despite all, the CVM common method still has a questionable diagnostic value to determine the growth spurt of the mandible.

Furthermore, studies have been conducted in the field of using artificial intelligence to estimate the CVM stages. These studies have limitations including the labeling of images by one observer and not taking into account the low inter and intra-observer agreement in the diagnosis of CVM stages, the lack of a second parameter to confirm and validate the bone maturation spurt, and not considering the uncertainty in the correlation between CVM and mandibular growth peak when using the common morphological method [9–14].

In this regard, this study aimed to estimate the developmental stage of the mandible incorporating artificial intelligence using the cervical vertebras in the lateral cephalometric images. It was our endeavor to achieve more agreement between these two parameters by not considering the usual CVM stages and instead by directly relating the cervical vertebras’ images with the growth slope of the mandible using annually taken lateral cephalograms.

## Materials and methods

### Dataset

The data used for deep learning were cropped images of cervical vertebras in lateral cephalometric radiographs, but for this purpose, the full cephalometric images must first be extracted and labeled according to the changes in mandibular length.

These images were obtained from the AAOF (American Association of Orthodontics Foundation) Craniofacial Growth Legacy Collection (aaoflegacycollection.org). The unique feature of this collection is the presence of serial lateral cephalometric images of the cases, which are mostly taken annually.

**Fig. 1:**
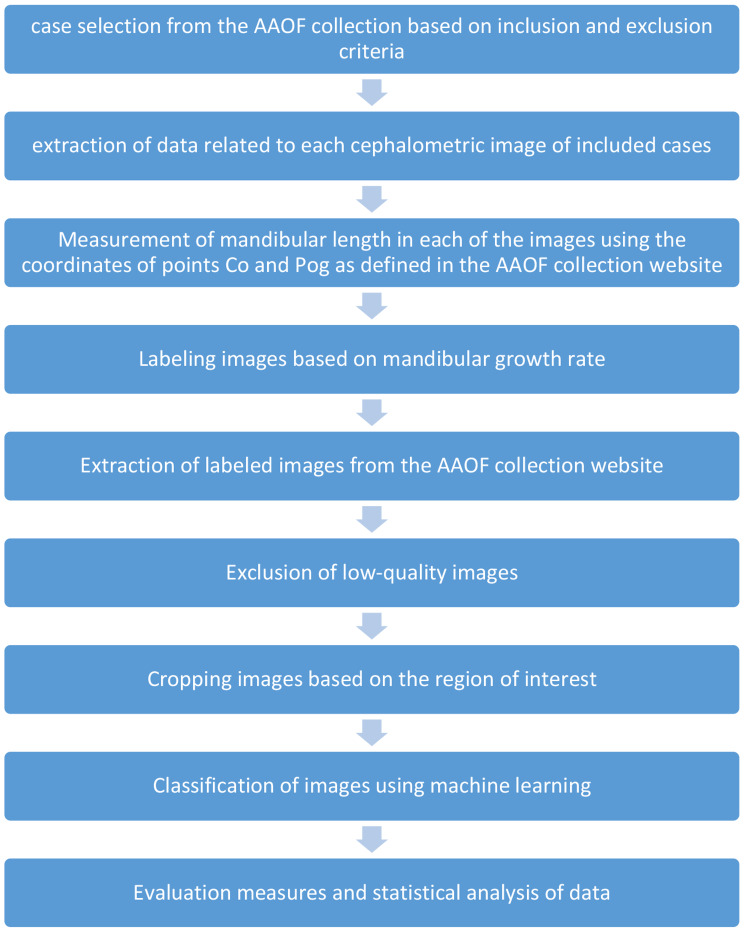
Study workflow chart

The study workflow is illustrated in Fig. 1.

In this study, we have used the data of these centers according to the inclusion and exclusion criteria mentioned below. The cases used in our research are a total of 200 people, including 108 women and 92 men.

### Inclusion criteria

1. The case in question has at least six lateral cephalometric graphs between the ages of 8 and 15.
2. The interval between two consecutive images should be between 9 and 18 months.
3. C2, C3, and C4 vertebras are visible in images.

### Exclusion criteria

4. 1. Skeletal class 3 cases.
5. 2. Syndromic cases or any deformity of the head or face.

The summary of the individuals’ information who entered the study is demonstrated in Table 1.

**Table 1:** Summary of information for individuals entered the study

Growth center	Number of participants	Females	Males	Skeletal class I relationship	Skeletal class II relationship
Bolton-Brush Growth center	32	11	21	6	26
Burlington Growth	36	17	19	9	27
Denver Growth	45	34	11	30	15
Forsyth Twin	9	4	5	6	3
Iowa Growth	8	4	4	8	0
Mathews Growth	30	20	10	15	15
Michigan Growth	17	7	10	10	7
Oregon Growth	23	11	12	6	17
Total number of participants	200	108	92	90	110

### Data preparation

**Fig. 2:**
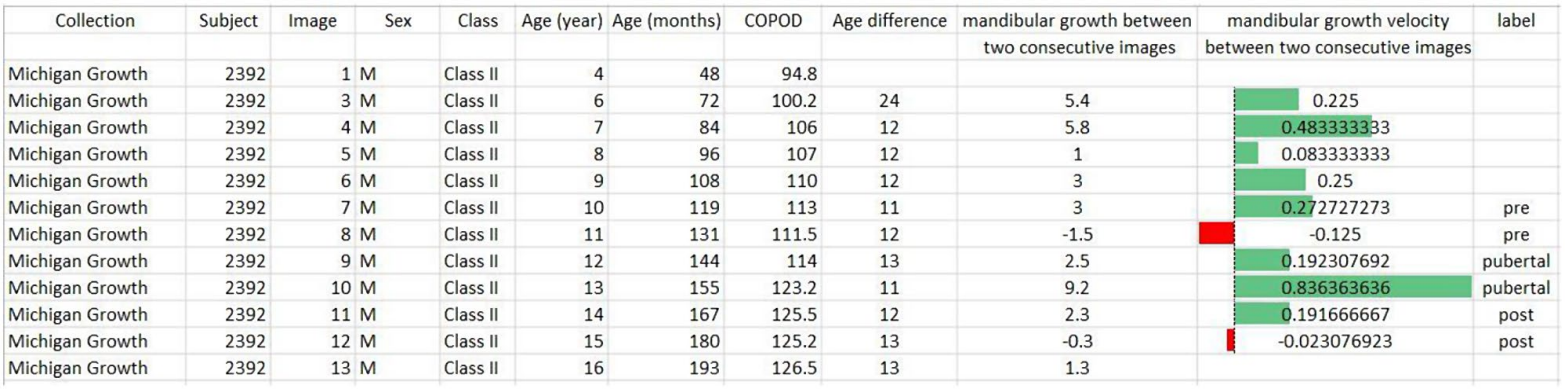
information table related to case number 2392 from the Michigan Growth Center

After selecting the cases, the information table for each person is prepared that includes the name of the growth center, the identification number registered for everyone in that center, gender and skeletal classification of the jaws, age of the person noted with the year and the month at the time of getting each graph and mandible growth rate between two consecutive graphs. An example of this table for case number 2392 from the Michigan Growth Center is shown in Fig. 2.

The gold standard of growth spurt is the rate of increase in mandibular length, and the images were labeled into three classes based on that. These stages are before the growth peak of puberty (pre-pubertal), during the growth peak of puberty (pubertal), and after the growth peak of puberty (post-pubertal) [3].

**Fig. 3:**
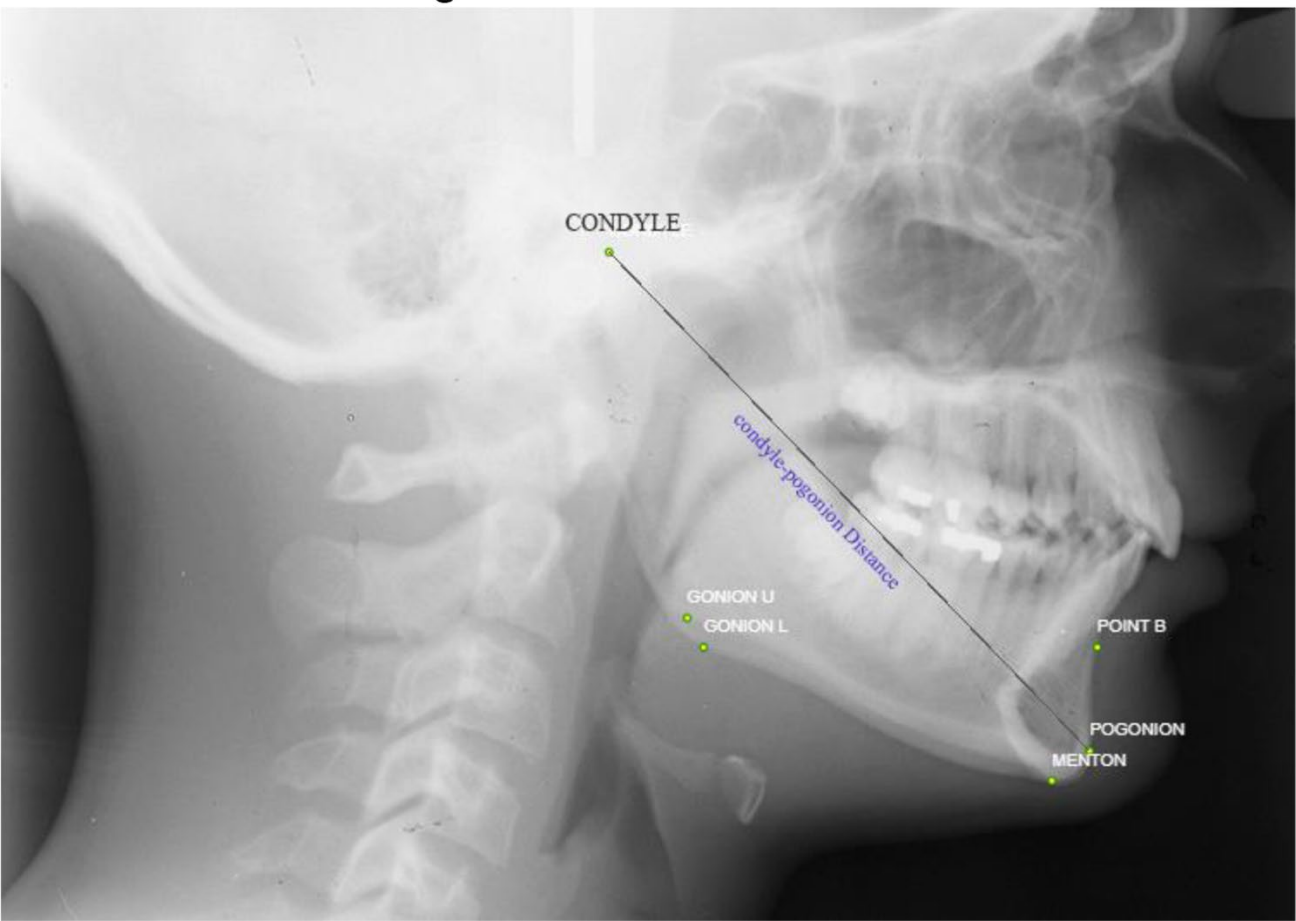
Measurement of mandibular length using the distance between two points Co and Pog on the lateral cephalometric graph of case number 2392 from the Michigan Growth Center

To calculate the length of the mandible, the distance between the two points of the Pogonion and Condyle was calculated according to Fig. 3. These points have been predetermined on the AAOF collection website. For measuring mandibular length, the coordinates provided on the website were used for mandibular length measurement.

In the next step, the growth rate of the mandible in each interval was obtained by dividing the increase in the length of the mandible between two consecutive graphs (in millimeters) by the time interval of two graphs (in months). The mandibular growth rate derived from each image indicates the mandibular growth status prior to capturing that cephalogram, obtained from the difference in mandibular length between the current and previous image. This data was recorded in the information table of each case.

To design a predictive artificial intelligence model, images were divided into training, validation and testing categories. After labeling the images, graphs related to each stage were adopted and 240 graphs were chosen for each stage, a total of 720 graphs. Some of the images did not have the necessary quality or suitable color intensity for use in neural networks, so they were excluded from the dataset, reducing the total number of images to 663. The total sets of images were divided into the ratio of 85 to 15 for training and testing, respectively, and then the training images were further divided into the ratio of 80 to 20 for training and validation.

The final structure of the dataset and its classes are described in Table 2.

**Table 2:** The final structure of the dataset

	Pre-pubertal	Pubertal	Post-pubertal
The number of images for the training phase	153	154	146
The number of images for the validation phase	38	39	37
The number of images for the test phase	32	32	32
The total number of images	223	225	215

**Fig. 4:**
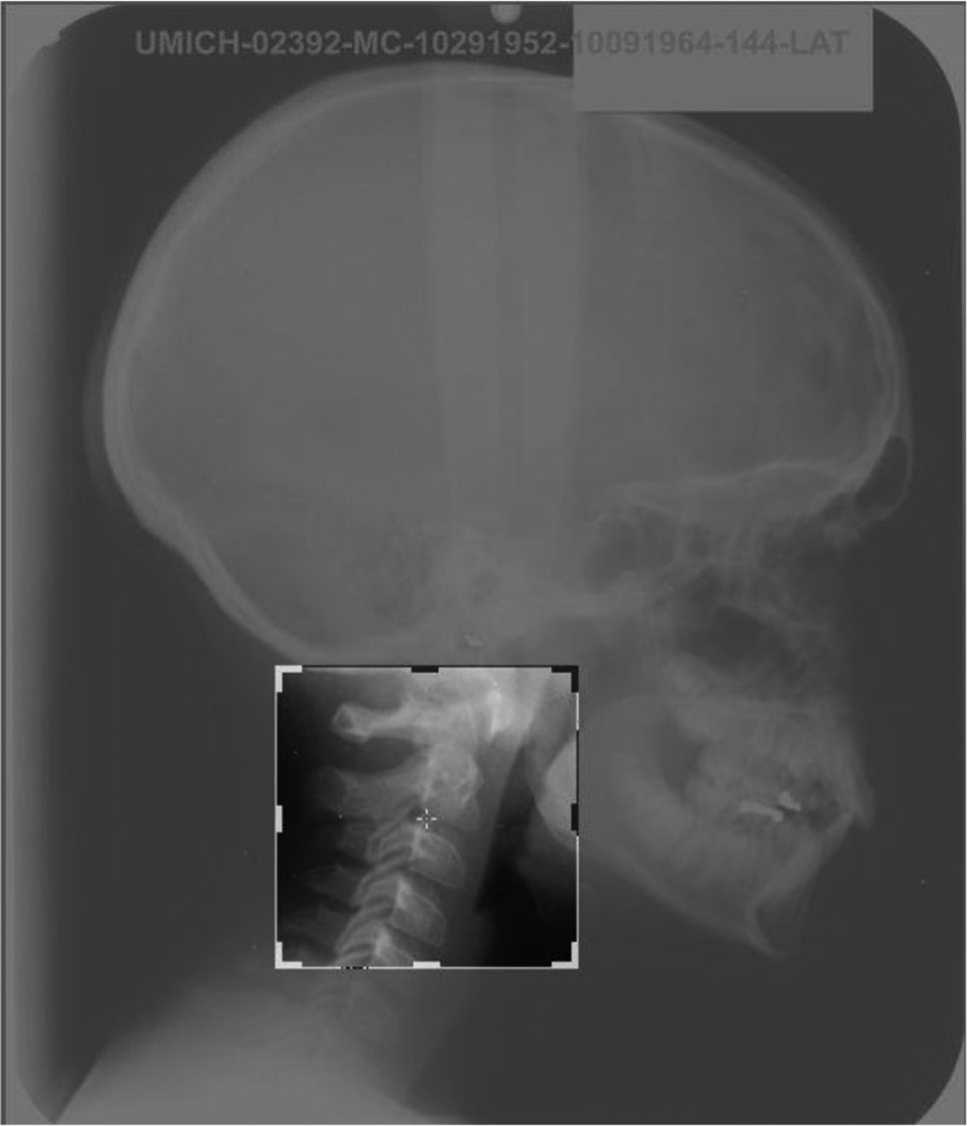
Region of Interest (ROI) related to case number 2392 from the Michigan Growth Center

Afterward, the part of the selected images related to the cervical vertebras was separated from the entire lateral cephalometric image as the region of interest(ROI). The upper limit of this area is the Basion point, the lowest point on the anterior margin of the foramen magnum at the base of the clivus, and its lower limit is the lower side of the C4 vertebra. An example of an ROI is shown in Fig. 4.

**Fig. 5:**
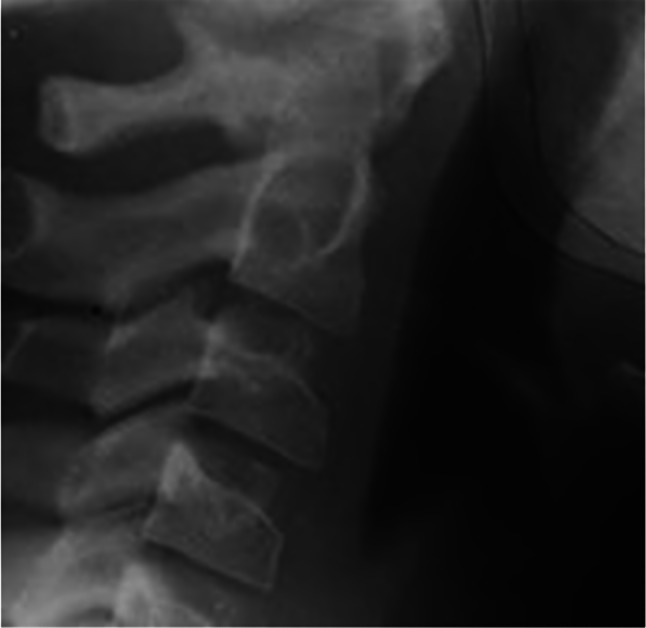
An example of an image related to pre-pubertal stage

**Fig. 6:**
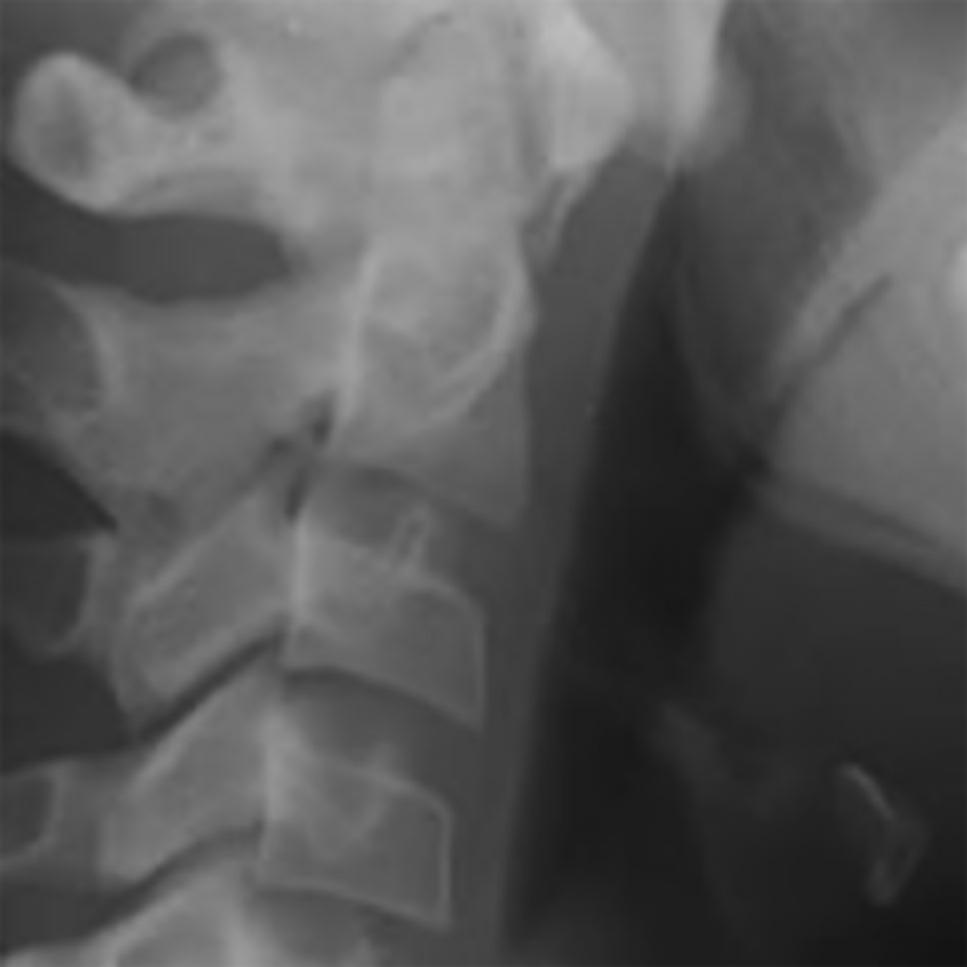
An example of an image related to pubertal stage

**Fig. 7:**
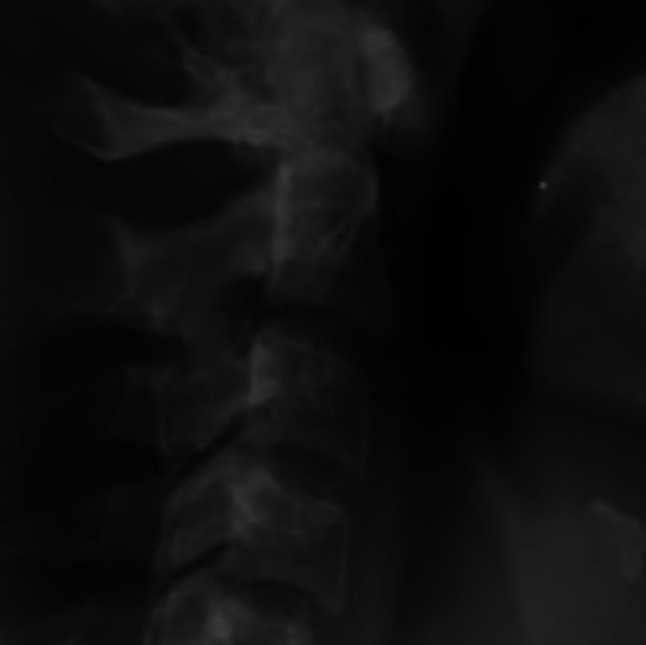
An example of an image related to post-pubertal stage

Examples of images from each class are shown in Figs. 5 and 6, and 7.

### Classification of images using machine learning

In this study, we used the Python programming language version 3.10.4 and the artificial intelligence libraries including Keras and TensorFlow version 2.9.1. We proposed a lightweight convolutional neural network (CNN) architecture for training and testing to overcome the problems with heavier models. No filters were used on the images, and the raw images were fed into the model for training. Only dimension reduction of the images was used to optimize the use of hardware resources and reduce the computational burden of unimportant image data. This also accelerated the training process. Batch normalization was used in the proposed architecture to improve model accuracy and reduce overfitting. Additionally, the technique of random dropout was used to reduce the risk of overfitting. Also, data augmentation was used to overcome the problem of data scarcity. Types of data augmentation for the ResNet network include rotating images up to an angle of 20 degrees, longitudinal and lateral displacement of the images by 10%, and magnification by a ratio of 10% on the images.

Different networks were evaluated to achieve higher accuracy and precision based on transfer learning for use in our small dataset. Two of them are discussed here, one specifically designed for this study and the other being ResNet-18.

The designed convolutional network for using in this study consists of three convolutional layers and 2 fully connected layers for classification. The input image size of 64*64 was used, which facilitates and speeds up the training process. The initial learning rate was set to the default value. The data was read using the ImageDataGenerator and fed to the model in batches.

Among the VGG architectures, the ResNet18 architecture was chosen. Makaremi and colleagues used ResNet18 for similar images with 6 classes due to its appropriate number of parameters for the dataset size [9].The ability to freeze layers was not used due to the difference between the radiology images in this study and the various colored images from ImageNet. The network was trained with random initial values for 200 epochs with a decreasing learning rate.

In this study, a computer with a core i7 1165G7 central processing unit, 16 gigabytes of random access memory (RAM), and an NVIDIA mx450 graphics card with 2 gigabytes of dedicated RAM was used to train the networks.

### Evaluation measures and statistical analysis of data

In this study, CVM was used as an indicator of mandibular growth, and the increase in mandibular length was considered the standard for the growth spurt. The agreement between the two indices was evaluated and reported using the following measures:

#### Sensitivity

the possibility that when the radiographic image is related to the time of one of the three developmental stages of the mandible, the artificial intelligence model also predicts the same stage.

#### Specificity

the possibility that when the radiographic image is not related to the time of one of the three developmental stages of the mandible, the artificial intelligence model does not predict the same stage.

#### Accuracy

the number of correct predictions compared to the total input samples.

#### Positive predictive value (PPV)

when the artificial intelligence model predicts one of the three developmental stages of the mandible, the input image should also be related to the same stage.

#### Negative predictive value (NPV)

when the artificial intelligence model does not predict the desired growth stage of the mandible, the input image is not related to the time of occurrence of the same stage.

To assess the level of agreement between the AI model’s predictions and mandibular growth stages, we also employed weighted kappa statistics. The interpretation of the kappa values was based on data according to Altman [15].

It should be noted that conducting this research was approved by the ethics committee of Tabriz University of Medical Sciences with ethical code ID IR.TBZMED.REC.1400.142.

The cephalometric images and related data are in the archives of the AAOF Craniofacial Growth Legacy Collection as it is provided free of charge for researchers.

## Results

**Fig. 8:**
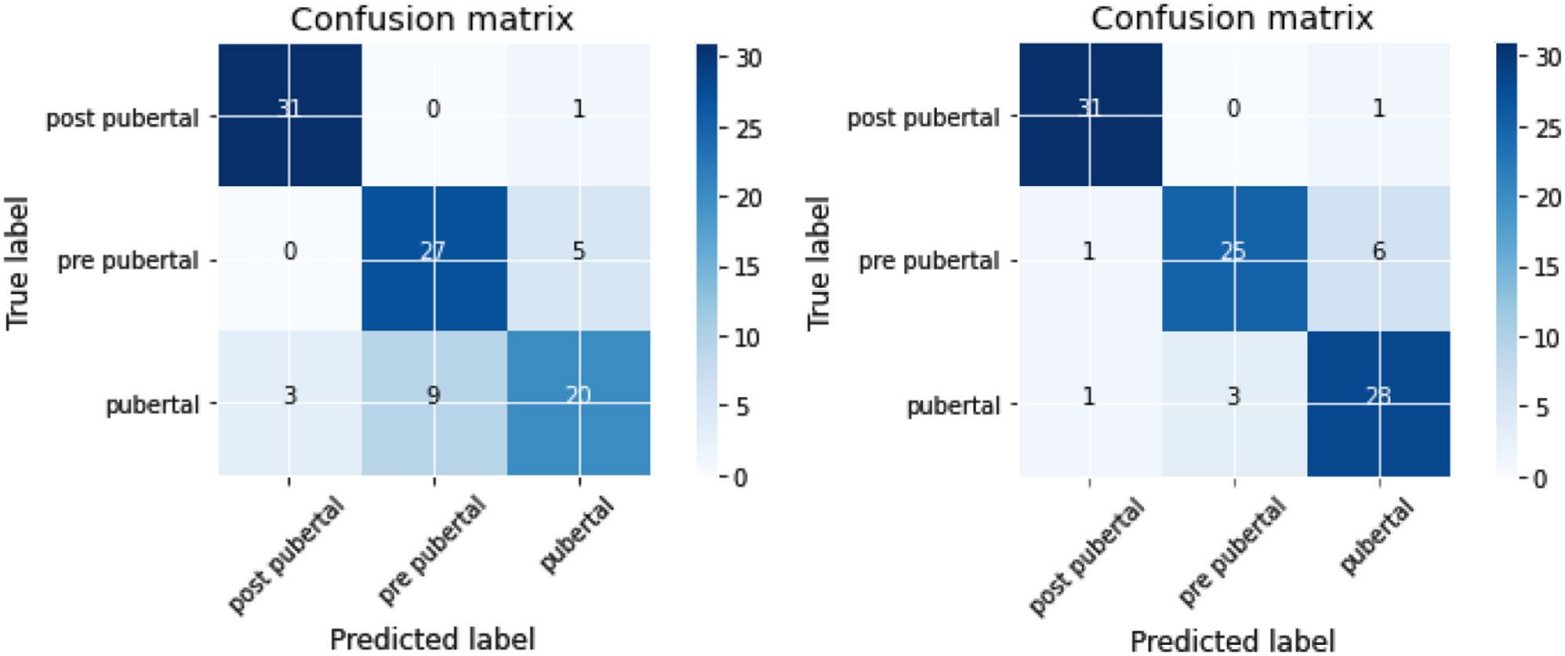
Confusion matrix related to designed convolutional neural network (left) and ResNet-18 model (right)

In the created model, the confusion matrices were obtained for the three classes by implementing different neural networks. Out of a total of 96 images in three classes in testing datasets, the convolutional neural network had 31 correct detections in the first class, 27 correct predictions in the second class, and 20 correct predictions in the third class, for a total of 78 correct detections. The ResNet network had 31, 25, and 28 correct detections for the first, second, and third classes, respectively, from the total of 96 images. These matrices are shown in Fig. 8.

The accuracies obtained using the designed convolutional neural network and ResNet-18 methods were 81% and 87.5%, respectively. The Weighted Kappa for the model’s performance in predicting the mandibular growth stage were approximately 0.7188 and 0.8125 for the designed convolutional neural network and the ResNet-18 model, respectively, indicating good and very good agreement according to Altman [15].

The results obtained by the designed convolutional neural network are presented in the Table 3.

**Table 3:** Results obtained from image classification using the designed convolutional neural network

	Sensitivity		Specificity		Positive predictive value (PPV)		Negative predictive value (NPV)		accuracy	
	value	rank	Value	rank	value	rank	value	rank	value	Rank
Pre pubertal	0.84375	2	0.859375	3	0.75	3	0.916667	2	0.854167	2
pubertal	0.625	3	0.90625	2	0.769231	2	0.828571	3	0.8125	3
Post pubertal	0.96875	1	0.953125	1	0.911765	1	0.983871	1	0.958333	1
	-		-		-		-		0.8125	

The results obtained by the ResNet-18 model are presented in the Table 4.

**Table 4:** Results obtained from image classification using ResNet-18 model

	Sensitivity		Specificity		Positive predictive value (PPV)		Negative predictive value (NPV)		accuracy	
	value	rank	Value	rank	value	rank	value	rank	value	Rank
Pre pubertal	0.78125	3	0.9375	2	0.862069	2	0.895522	3	0.885417	3
pubertal	0.875	2	0.90625	3	0.823529	3	0.935484	2	0.895833	2
Post pubertal	0.96875	1	0.96875	1	0.939394	1	0.984127	1	0.96875	1
	-		-		-		-		0.875	

**Fig. 9:**
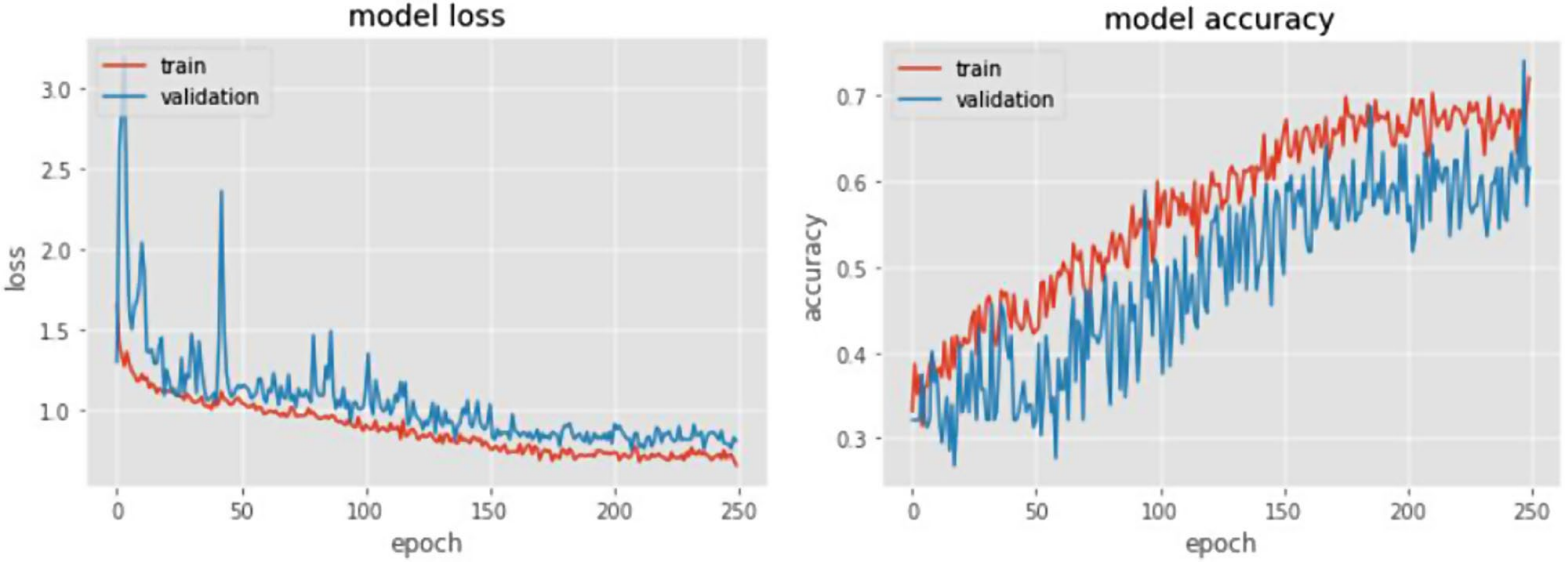
The loss function (left) and the accuracy (right) of the designed convolutional neural network model

**Fig. 10:**
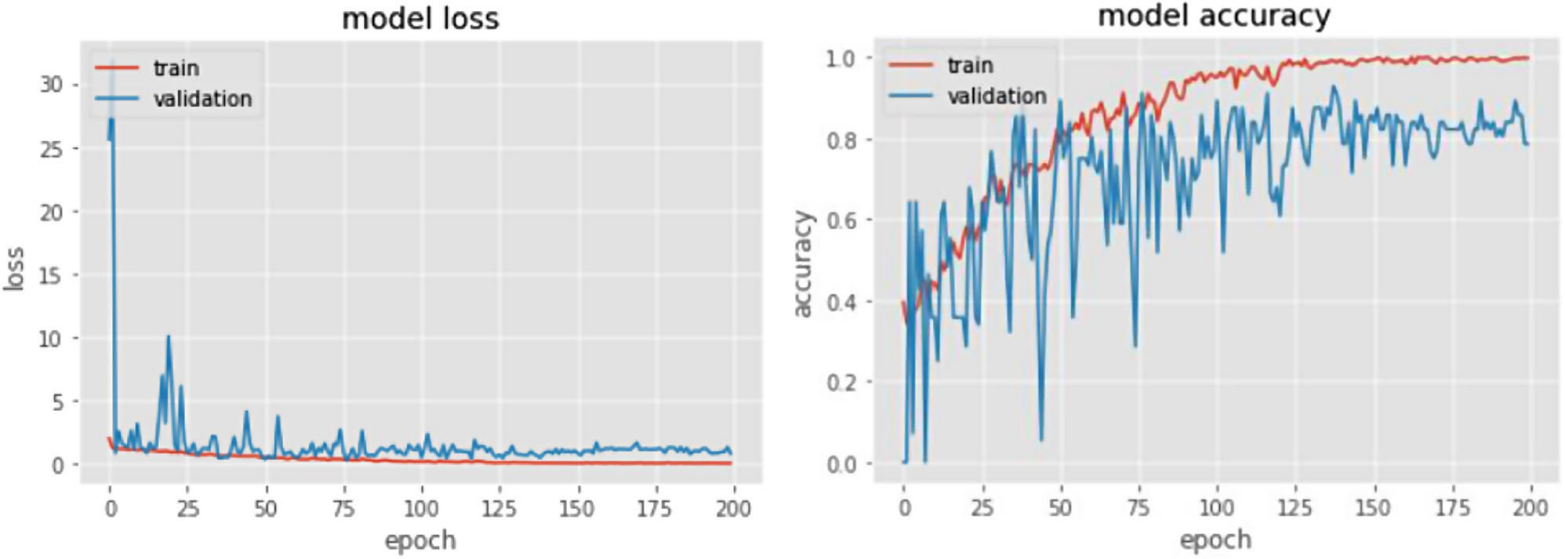
The loss function (left) and the accuracy (right) of the designed convolutional neural network model

The loss function and the accuracy diagram of the designed convolutional neural network model and ResNet-18 model are shown in Figs. 9 and 10, respectively.

**Fig. 11:**
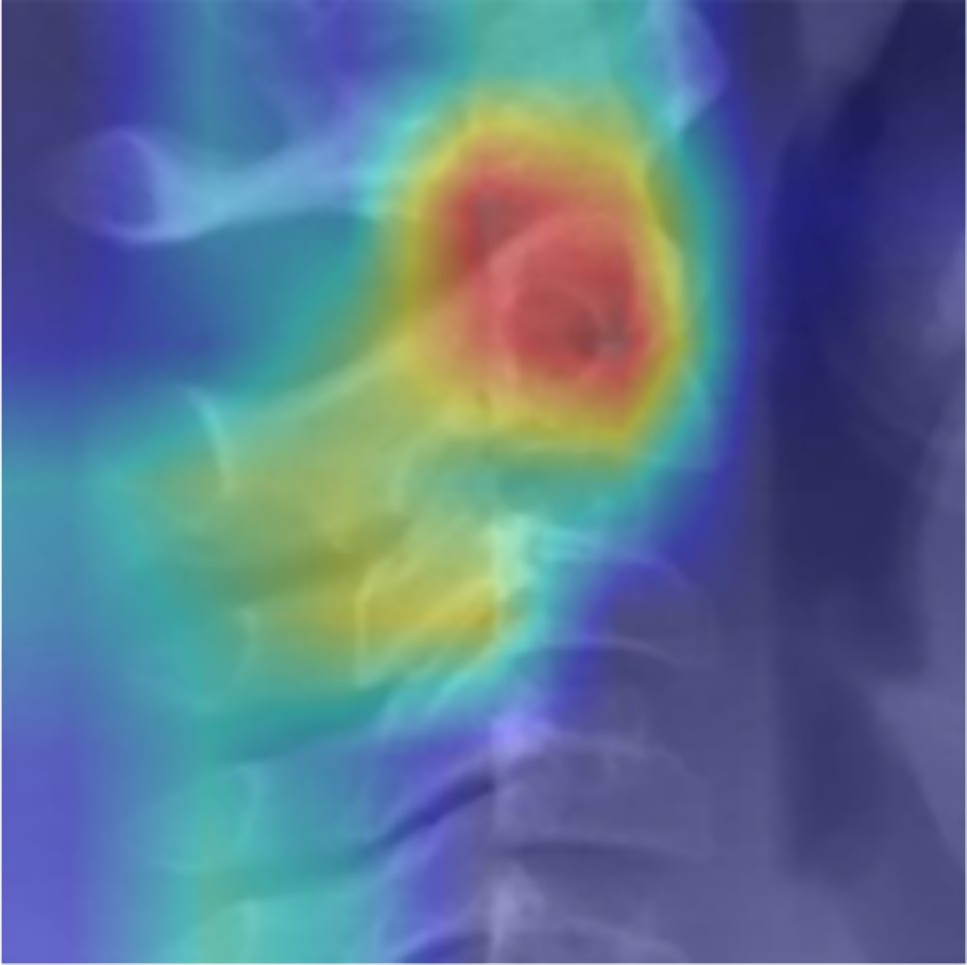
Heat map related to pre pubertal stage

**Fig. 12:**
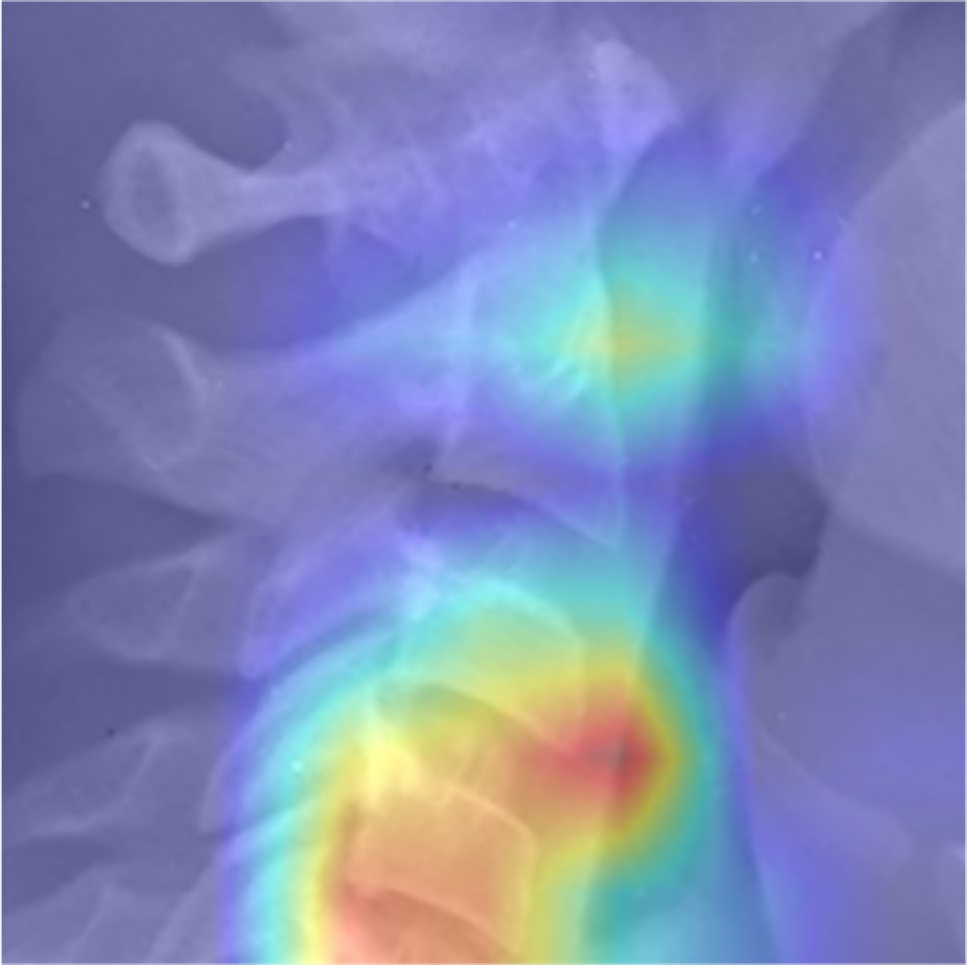
Heat map related to pubertal stage

**Fig. 13:**
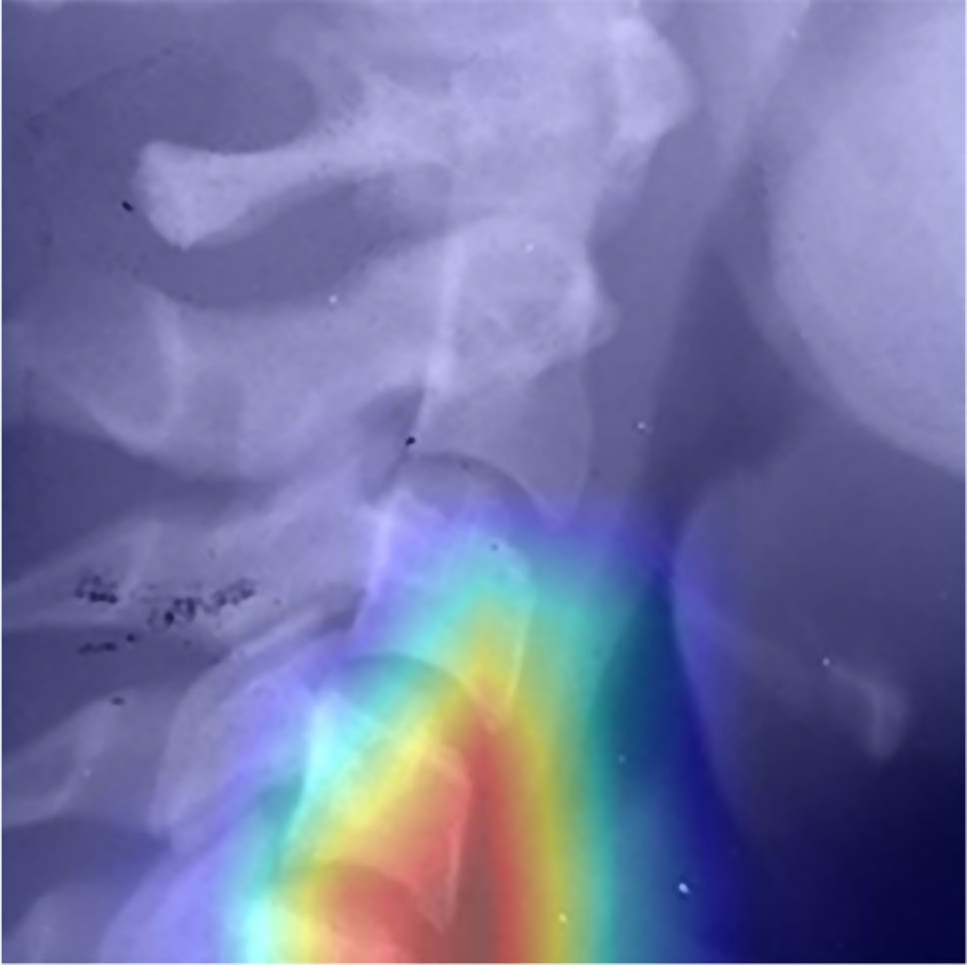
Heat map related to post pubertal stage

Heat maps obtained from the model for each stage are presented in Figs. 11, 12 and 13. These figures illustrate the focal points of the artificial intelligence model for extracting common features present in the images of each classification [16].

## Discussion

In this study, patients with skeletal Class III malocclusion were excluded at the beginning. This decision was based on evidence suggesting that these individuals exhibit an atypical growth pattern, longer and more extensive growth compared to those in Class I and Class II. Furthermore, determining the mandibular growth stage using CVM is not clinically applicable in the treatment of growth modification in Class III patients [17].

The ResNet-18 model had better performance in almost all measurements compared to the designed convolutional neural network, except for sensitivity and NPV in the pubertal stage. Also, both artificial intelligence models used in this study had the highest accuracy in identifying images related to the post-pubertal stage.

The use of artificial intelligence technology in detecting the maturation stages of cervical vertebras is one of the applications of artificial intelligence in orthodontics. Considering the importance of timing in orthodontic treatment and the methods of determining maturity stage, as well as the benefits of using artificial intelligence in the diagnostic stages. Artificial intelligence models as a diagnostic aid can be used to improve treatment planning by making ease of work and increasing the accuracy of diagnosis.

Initially, software programs were developed for semi-automatic detection of cervical vertebral maturation stages. These programs required manual determination of anatomical points, following which the software predicted the maturation stage. In the study of Baptista et al. landmarks were identified on the cephalometric images and semi-automatic software was designed according to the obtained patterns. The results of the study showed that the proposed semi-automatic classification method can help orthodontists in identifying the CVM stage [10].

In another study, Santiago et al. tried to design software that can quantitatively analyze the maturation of cervical vertebras through a logistic regression model. As a result of the study, the quantitative method implemented through the software had reproducibility. The model used in this research, which was a combination of four parameters measured on the vertebral body, plus age and sex, showed satisfactory prediction [11].

With the advancement of artificial intelligence technology, more research was performed to further automate this process. A study by Kok et al. was conducted to design and compare 7 types of artificial intelligence algorithms in detecting cervical vertebral maturation stages. In this study, first, the cephalometric images were labeled by an orthodontist based on the 6 stages of CVM maturity, then 19 reference points were determined on the C2, C3, and C4 vertebrae, and measurements were made. By receiving these measurements as input data, the algorithms delivered the estimated maturity stage as an output, and then these outputs were compared with the real CVM maturity stage determined by the orthodontist to compare the diagnostic accuracy. According to this study, the artificial neural network (ANN) algorithm has performed better than others [12].

Other similar research has been done by Amasya et al. using 26 anatomical points and measurements as input data. According to this study, the best results are obtained with the ANN algorithm [14].

Another study was conducted in France to process the cervical vertebras images in a fully automatic manner using deep learning and the CNN method. In this research, at first, images were labeled by a radiologist to 6 stages of CVM according to the Bacceti method. The researchers have used a five-layer DL-CNN classification method. They suggested that it is suitable for the mentioned data and study. To train the proposed model, in the first step, they used 300 labeled images for training, 200 images for validation, and finally 100 images for testing. In the following, they increased the number of training data sets to 600, then to 900, and finally to 1870 images. Most importantly, this study has shown that an artificial intelligence model can detect the maturation stages of the cervical vertebras directly from the lateral cephalometric radiograph itself with an average accuracy of 90% [9]. The limitations of this study are the labeling of the photos by a radiologist and the lack of radiography of the hand wrist or another growth indicator to confirm and validate the results.

As can be seen, in most of the previous studies, the measurements obtained from the images are included in the study instead of the images themselves, and for this reason, they are different from the study we did. Only the study by Makaremi et al. used cephalometric images as the model input, but also that study itself differs from ours’ in areas such as objectives and criteria for labeling images.

In previous studies, cephalometric images have been classified based on the shape of the cervical vertebrae, and in fact, the output and goal of the previous studies are to estimate the developmental stage of the cervical vertebras. While in our study, the images are labeled based on the growth rate of the mandible and not the shape of the cervical vertebras. On the other hand, supervised artificial intelligence methods extract common features in the images of a category in the learning phase and then in the next step, predict the corresponding category of the input images.

The important point is that the purpose of determining the CVM stages in orthodontics is predicting the developmental status of the mandible, and the diagnostic value of the conventional CVM method to determine the growth spurt of the mandible is room for question.

Studies on the relationship between CVM and mandibular growth spurt show contradictory results. A systematic review of the predictability of the CVM discovered that most researches have methodological shortcomings [18]. The small sample size is one of the flaws in some studies that assess the CVM diagnostic value [19–21]. However, some studies indicated that the CVM method shows good results in determining the growth peak of the mandible. A study was conducted in 2016 by Perinetti et al., to investigate the diagnostic value of the CVM method and height growth rate in determining mandibular growth spurt. The diagnostic accuracy of the CVM method in determining the growth spurt in the mandibular length in the age range of 11–12 years, 12–13 years, and 13–14 years were 0.74, 0.71, and 0.67 respectively [22].

Another study was conducted by Mellion et al. on the data of 100 cases and investigating the relationship between different growth indices with height maturity and mandibular growth spurt. This study shows the concordance of the interval between the third and fourth stages of the CVM method and the growth spurt of the mandibular height is 0.33 in girls and 0.52 in boys, which indicates that The CVM method alone cannot accurately estimate the growth spurt [23].

Also, the validity of CVM in the evaluation of craniofacial growth has been questioned [19, 20, 24]. High levels of reproducibility are informed in some studies, although they use tracings of lateral cephalograms instead of actual images to determine the CVM stages [25].In another study Rainey et al. the reliability of the CVM method and discovered significant intra- and interobserver agreements, inferring that the conventional approach of CVM classification is reliable [26]. In a study conducted by Predko-Engel et el., inter-observer and intra-observer reproducibility was investigated for the CVM method. Based on that, the average repeatability in one observer was 52% and 42% between observers [24]. These contradictory results show that determining the different stages of CVM by the observer is not a reliable method. In the previous research, as mentioned above, the labeling of images was done by an observer as the reference standard, and no other growth index was used.

According to the discussed evidence, even if an artificial intelligence model can predict the development stage of the cervical vertebras with an accuracy of more than 90%, it does not mean it is capable of predicting the mandibular growth spurt with high accuracy. While the artificial intelligence models designed in this study have an accuracy of 87.5% in estimating the growth status of the mandible, which is comparable, even better, to the usual CVM method for determining the growth spurt of the mandible.

Furthermore, it appears in heat maps that the artificial intelligence model follows a certain pattern of cephalocaudal growth in the images corresponding to each classification. Additionally, in the pre-pubertal images, aside from the vertebrae, the presence of spinous processes is also among the focal points, which may indicate the existence of hidden features for staging growth in this area.

### Limitations and suggestions

The low quality of the images was a limitation in our study that can affect the accuracy of the designed model. Moreover, previous studies expressed that increasing the number of images, if the number of images in different groups was equal and homogeneous, enhances the accuracy of the artificial intelligence model. it is suggested to use more images in the following studies [9].

Considering the difference in agreement between the CVM method and the mandibular growth spurt in different genders and ages, as well as the possibility of influence of the facial growth pattern on this relationship, it is suggested to categorize people by considering these variables and train the artificial intelligence model based on these categories [23].

## Conclusion

The artificial intelligence model designed in this study has the ability to be used as a diagnostic aid tool along with other growth indicators to estimate mandible growth status. This model had the highest accuracy in estimating pre-pubertal and pubertal stages. Therefore, it is more efficient to determine the occurrence of a mandibular growth spurt before or during puberty.

## Data Availability

The datasets used and/or analysed during the current study are available from the corresponding author on reasonable request.

## Abbreviations

CVM: Cervical vertebral maturation
AI: Artificial intelligence
CNN: Convolutional neural network
NPV: Negative predictive value
PPV: Positive predictive value
AAOF: American Association of Orthodontics Foundation
ANN: Artificial Neural Network
ROI: Region of Interest
